# Neuroendocrine immunomodulation network dysfunction in SAMP8 mice and PrP-hAβPPswe/PS1^ΔE9^ mice: potential mechanism underlying cognitive impairment

**DOI:** 10.18632/oncotarget.8453

**Published:** 2016-03-28

**Authors:** Jian-hui Wang, Xiao-rui Cheng, Xiao-rui Zhang, Tong-xing Wang, Wen-jian Xu, Fei Li, Feng Liu, Jun-ping Cheng, Xiao-chen Bo, Sheng-qi Wang, Wen-xia Zhou, Yong-xiang Zhang

**Affiliations:** ^1^ Department of Neuroimmunopharmacology, Beijing Institute of Pharmacology and Toxicology, Beijing, China; ^2^ State Key Laboratory of Toxicology and Medical Countermeasures, Beijing, China; ^3^ Department of Biotechnology, Beijing Institute of Radiation Medicine, Beijing, China

**Keywords:** Alzheimer's disease, neuroendocrine immunomodulation, Senescence-accelerated mouse prone 8 strain (SAMP8), PrPhAβPPswe/PS1ΔE9 (APP/PS1) mice, learning and memory, Gerotarget

## Abstract

Senescence-accelerated mouse prone 8 strain (SAMP8) and PrP-hAβPPswe/PS1^ΔE9^ (APP/PS1) mice are classic animal models of sporadic Alzheimer's disease and familial AD respectively. Our study showed that object recognition memory, spatial learning and memory, active and passive avoidance were deteriorated and neuroendocrine immunomodulation (NIM) network was imbalance in SAMP8 and APP/PS1 mice. SAMP8 and APP/PS1 mice had their own specific phenotype of cognition, neuroendocrine, immune and NIM molecular network. The endocrine hormone corticosterone, luteinizing hormone and follicle-stimulating hormone, chemotactic factor monocyte chemotactic protein-1, macrophage inflammatory protein-1β, regulated upon activation normal T cell expressed and secreted factor and eotaxin, pro-inflammatory factor interleukin-23, and the Th1 cell acting as cell immunity accounted for cognitive deficiencies in SAMP8 mice, while adrenocorticotropic hormone and gonadotropin-releasing hormone, colony stimulating factor granulocyte colony stimulating factor, and Th2 cell acting as humoral immunity in APP/PS1 mice. On the pathway level, chemokine signaling and T cell receptor signaling pathway played the key role in cognition impairments of two models, while cytokine-cytokine receptor interaction and natural killer cell mediated cytotoxicity were more important in cognitive deterioration of SAMP8 mice than APP/PS1 mice. This mechanisms of NIM network underlying cognitive impairment is significant for further understanding the pathogenesis of AD and can provide useful information for development of AD therapeutic drug.

## INTRODUCTION

Alzheimer's disease (AD) is a progressive, irreversible and age-related neurodegenerative disease. AD mainly includes sporadic AD (SAD) which occurs in patients aged 65 years or older and familial AD (FAD) which accounts for early onset autosomal dominant forms. There is no treatment prevent, halt, or reverse AD. Besides the design problems and possible strategies including the aspects of patients, drugs, outcome measurements, trial protocol, and optimization of resources [1], the possible reason of regretful results of these disease-modifying drugs could be poor understanding and methodology in AD animal studies [2, 3].

PrP-hAβPPswe/PS1^ΔE9^ (APP/PS1) mice and senescence-accelerated mouse prone 8 strain (SAMP8) are classic animal models of FAD and SAD respectively. The direct reason causing learning and memory impairment in APP/PS1 mice is considered the overexpression of the hAPP encoding gene with Swedish mutation together with the mutant PS1 gene, which are combined to reach elevated amyloid-β (Αβ) levels [4]. But the behavioral defects do not show a synchronization with pathological changes [5, 6] and the mechanisms responsible for the AD-like behaviors of APP/PS1 mice may be associated with the presences of intensive gliosis [7–9], tau-positive neuritis [10], and dystrophic excitatory synaptic boutons [11] with progressive amyloidosis. While, the direct reason causing learning and memory impairment in SAMP8 mice is related to accelerated ageing. But it has also been reported that the mechanisms of cognitive impairments in SAMP8 mice may be related with neuronal degeneration [12], inflammatory-amyloid cycle driven by oxidative stress [13], age-related decrease in serum androgen and estrogen levels [14, 15], elevated triggering receptor expressed on myeloid cells 2 (TREM2) [16], increased expression of pro-inflammatory cytokines [17]. Therefore, the mechanisms of cognitive impairment in SAMP8 and APP/PS1 mice remain elusive and unclear in multi-dimension and integrated system.

Neuroendocrine immunomodulation (NIM) network plays an important role in the process of adaptation, homeostasis, and defense against the factors of a hostile environment [18]. The balance of NIM network maintains the whole physiological homeostasis of body and normal physiological process. Therefore, in this study, we investigated the underpinnings of learning and memory deficits in both AD animal models, SAMP8 and APP/PS1 mice, based on NIM network. We first comprehensively examined the phenotypes of cognition, neuroendocrine, immune and neuroendocrine immunomodulation molecular network in both AD model. Then we identified the key molecules in NIM network and cellular pathways contributing to cognitive impairment in SAMP8 and APP/PS1 mice using multiple linear regressions analysis and network fingerprint analysis.

## RESULTS

### The cognitive impairment in SAMP8 and APP/PS1 mice

**Figure 1:**
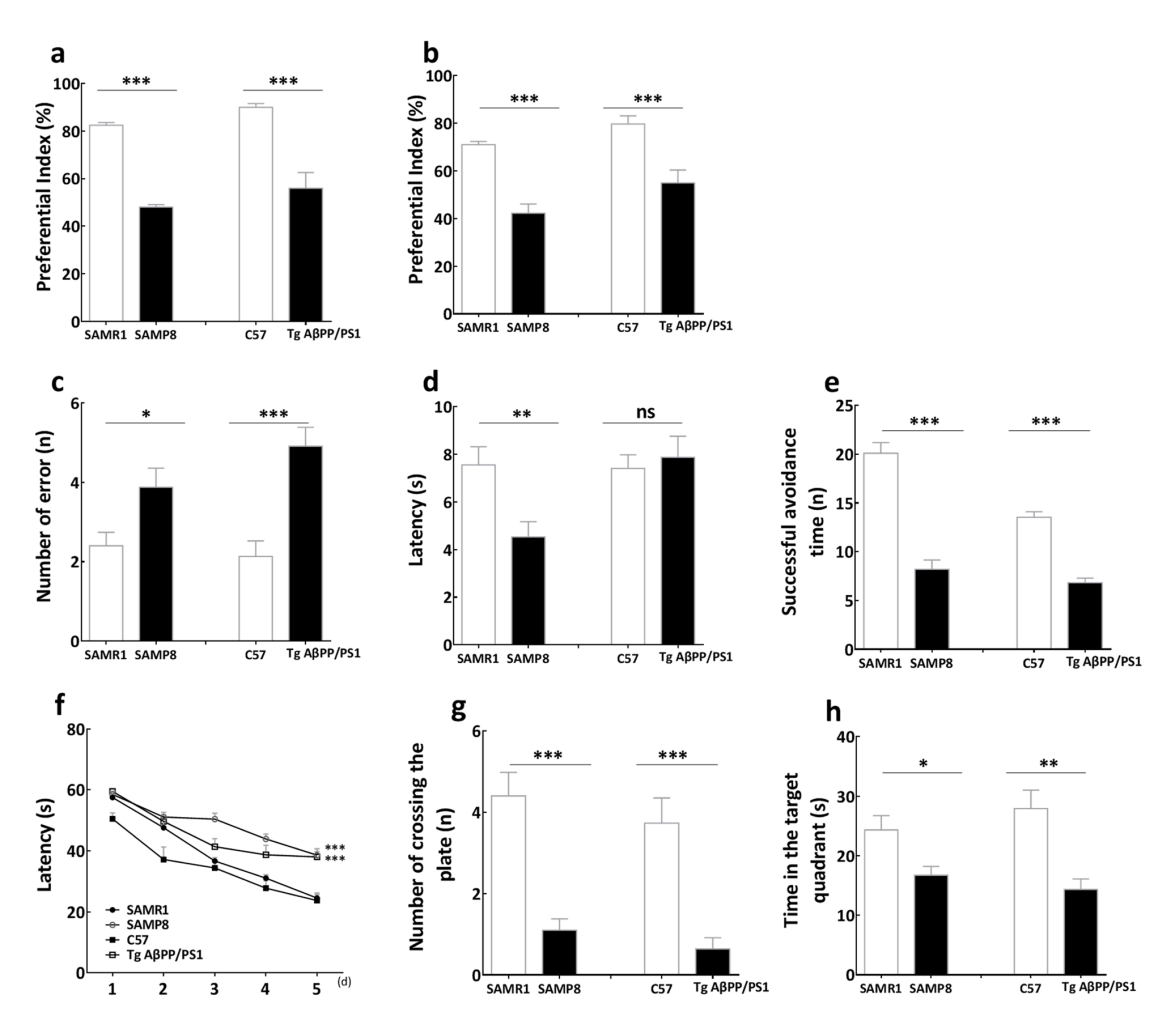
The behavior of learning and memory in SAMP8 mice and PrP-hAβPPswe/PS1^ΔE9^ mice The preferential index in novel object recognition test showed that both SAMP8 mice and PrP-hAβPPswe/PS1^ΔE9^ mice were deficit in the short **a.** and long **b.** term object recognition memory respectively. Comparing with SAMR1 mice and C57 mice, the number of error of SAMP8 mice and PrP-hAβPPswe/PS1^ΔE9^ mice **c.** increased in step-down test respectively, while the escape latency reduced in SAMP8 rather than PrP-hAβPPswe/PS1^ΔE9^ mice **d.** indicated that the passive avoidance response was deficient in SAMP8 mice and PrP-hAβPPswe/PS1^ΔE9^ mice. The successful avoidance times in shuttle-box test **e.** indicated that the active avoidance response was deteriorated in SAMP8 and PrP-hAβPPswe/PS1^ΔE9^ mice. The escape latencies in the learning task **f.**, the number of crossing platform **g.** and time in the target quadrant **h.** in the probe trial of Morris water maze test indicated that the spatial learning and memory ability was deficient in SAMP8 mice and PrP-hAβPPswe/PS1^ΔE9^ mice. The “ns” is that there is no statistical significant difference, *P < 0.05, **P < 0.01, ^***^P < 0.001, compared with control mice. Data represent mean ± SEM, *n* = 10~15, unpaired Student *T*-test.

The novel object recognition test was used to investigate the object recognition memory. The preferential index at 1 and 24 hours after training indicated the short and long term object recognition memory respectively. Results showed that the preferential index significantly decreased at 1 and 24 hours after training in SAMP8 mice (Figure 1a), as well as in APP/PS1 mice (Figure 1b), compared with their control SAMR1 and C57BL/6J (C57) mice, respectively. This indicated that both SAMP8 mice and APP/PS1 mice were deficit in the short and long term object recognition memory respectively.

The step down test was used to determine passive avoidance of SAMP8 mice and APP/PS1 mice. Results showed that SAMP8 mice had a significant increased number of errors (Figure 1c) and a decreased in the latency of reactions compared with the SAMR1 mice in step down test (Figure 1d). Likewise, comparing with C57 mice, the number of errors increased in APP/PS1 mice (Figure 1c). This indicated that there was impairment of passive avoidance in SAMP8 mice and APP/PS1 mice.

The shuttle box test was used to assess the active avoidance of SAMP8 mice and APP/PS1 mice. Results showed that the successful avoidance times significantly decreased in SAMP8 mice and APP/PS1 mice compared with SAMR1 and C57 mice respectively (Figure 1e). This indicated that the active avoidance response was deteriorated in SAMP8 and APP/PS1 mice.

The Morris water maze test was used to examine ability of spatial learning and memory. Results showed that SAMP8 mice and APP/PS1 mice exhibited longer retention latencies than SAMR1 and C57 mice respectively in the learning task (Figure 1f). This result indicated that the impairment of spatial learning occurs in the SAMP8 mice and APP/PS1 mice. The number of crossing platform (Figure 1g) was less and the time in the target quadrant (Figure 1h) was shorter than SAMR1 and C57 mice respectively in the probe trial of Morris water maze test. This indicated that the spatial memory ability was deficient in SAMP8 mice and APP/PS1 mice.

### The abnormality of endocrine hormone and cytokine in SAMP8 mice and APP/PS1 mice

In order to investigate the mode of hypothalamic-pituitary-adrenal (HPA) and hypothalamic-pituitary-gonadal (HPG) axis in SAMP8 mice and APP/PS1 mice, the concentration of gonadotropin-releasing hormone (GnRH) and concentrations of corticotropin releasing hormone (CRH) in hypothalamuses, adrenocorticotropic hormone (ACTH), follicle-stimulating hormone (FSH) and luteinizing hormone (LH) in pituitaries were measured by radioimmunoassay. The level of testosterone (T) and corticosterone (CORT) in plasmas were measured by immunochemiluminescence assay and ELISA respectively. Results showed that CRH, ACTH, and CORT were significantly increased in SAMP8 and APP/PS1 mice compared with SAMR1 and C57 mice respectively. Moreover, the concentration of CRH in SAMP8 mice was more double than that in SAMR1 mice. This indicated that HPA axis was hysteric in SAMP8 mice and APP/PS1 mice, and was more noticeable in SAMP8. The level of GnRH, FSH and LH were significant increase and T were decrease in SAMP8 and APP/PS1 mice compared with SAMR1 and C57 mice respectively. The concentration of LH in SAMP8 mice was more double than that in SAMR1 mice, T was less half of that in SAMR1 mice. This indicated HPG axis was depressed in SAMP8 mice and APP/PS1 mice, and was more remarkable in SAMP8. These date signified HPA and HPG axis were disorder in SAMP8 mice and APP/PS1 mice (Table 1).

**Table 1:** The endocrine hormone and cytokines of SAMP8 mice and PrP-hAβPPswe/PS1^ΔE^^9^ mice

endocrine hormone/cytokines	SAMR1(pg/mg or mIU/mg)	SAMP8(pg/mg or mIU/mg)	Ratio(SAMP8:SAMR1)	C57(pg/mg or mIU/mg)	Tg AβPP/PS1(pg/mg or mIU/mg)	Ratio(Tg AβPP/PS1:C57)
*Hypothalamic-pituitary-adrenal (HPA) axis*						
Corticotropin releasing hormone (CRH)	0.55±0.21	1.13±0.46**	2.08	73.12±19.05	101.17±17.04^***^	1.38
Adrenocorticotropic hormone (ACTH)	0.76±0.12	1.06±0.42**	1.40	54.63±19.49	98.88±45.98^***^	1.81
Corticosterone (CORT)	198.31±76.59	390.82±100.09**	1.97	234.5±54.95	447.07±129.47^***^	1.91
*Hypothalamus-pituitary-gonadal (HPG) axis*						
Gonadotropin-releasing hormone (GnRH)	1.35±0.21	2.38±0.81**	1.76	4.06±0.97	5.34±1.01^***^	1.32
Follicle-stimulating hormone (FSH)	0.2±0.11	0.31±0.08*	1.50	0.19±0.06	0.37±0.12^***^	1.91
Luteinizing hormone (LH)	0.4±0.16	0.9±0.29^***^	2.25	0.49±0.17	0.89±0.24^***^	1.81
Testosterone (T)	11.75±3.51	5.65±1.36^***^	0.48	8.03±4.51	5.63±2.3*	0.70
*Proinflammatory factor*						
*Interleukin*						
IL-1β	46.82±6.92	92.23±5.74***	1.97	29.39±6.89	76.22±11.59***	2.59
IL-2	27.2±5.01	58.3±6.1***	2.14	30.62±11.4	49.2±8.23***	1.61
IL-6	51.35±4.6	68.49±6.39***	1.33	111.83±14.71	136.56±12.33***	1.22
IL-23	82.53±9.45	104.64±8.9***	1.27	86.32±10.14	104.9±6.89***	1.22
IL-17	18.21±5.68	22.62±4.51	1.24	17.06±2.81	20.51±2.67**	1.20
*Colony stimulating factor*						
GM-CSF	17.62±3.11	23.9±1.14***	1.36	25.62±3.49	38±3.19***	1.48
*Interferon*						
INF-γ	67.44±4.35	116.19±12.6***	1.72	20.65±3.41	18.37±2.23	0.89
*Tumor necrosis factor*						
TNF-α	77.31±5.24	122.9±8.93***	1.59	35.95±14.89	78.82±14.35***	2.19
TNF-β	78.44±8.82	131.63±8.17***	1.68	73.44±9.81	149.78±9.61***	2.04
*Chemotactic factor*						
RANTES	31.59±5.33	42.51±6.21***	1.35	33±6.02	36.05±5.22	1.09
eotaxin	306.11±17.42	427.42±14.07***	1.40	337.06±74.9	465.84±40.03***	1.38
MCP-1	103.34±13.17	74.83±12.83***	0.72	117.02±12.66	119.2±13.43	1.02
MIP-1β	14.57±3.26	15.05±2.6	1.03	7.22±2.43	7.77±1.71	1.08
*Antiinflammatory factor*						
IL-4	45.67±6.32	36.36±5.84**	0.80	42.95±6.01	36.85±3.05**	0.86
IL-5	30.5±4.73	19.09±6.22***	0.63	25.03±12.87	27.9±11.12	1.11
IL-10	30.12±6.41	20.89±7.05***	0.78	no data	no data	no data
G-CSF	60.84±4.67	61.9±5.92	1.02	78.81±14.22	49.55±13.89***	0.63

Concentrations (pg/mg) of gonadotropin-releasing hormone (GnRH) and corticotropin releasing hormone (CRH) in hypothalamus, concentrations (mIU/mg) of follicle-stimulating hormone (FSH), luteinizing hormone (LH) and concentrations (pg/mg) of adrenocorticotropic hormone (ACTH) in pituitaries were measured by radioimmunoassary. Concentrations (pg/mg) of testosterone (T) in the plasma were measured using immunochemiluminescence assay. The concentrations (pg/mg) of corticosterone (CORT) were measured by enzyme linked immunosorbent assay (ELISA). For HPA axis, concentrations of CRH, ATCH and CORT were increased in SAMP8 mice and PrP-hAβPPswe/PS1^ΔE9^ mice compared with SAMR1 mice and C57 mice respectively. For HPG axis, concentrations of GnRH, FSH and LH were increased in SAMP8 mice and PrPhAβPPswe/PS1^ΔE9^ mice, and concentrations of T were decreased compared with SAMR1 mice and C57 mice respectively. Concentrations (pg/mL) of cytokines in the blood plasma were detected using Luminex^®^ X-MAP^®^ technology. Comparing with SAMR1 mice and C57 mice respectively, proinflammatory factor interleukin-1β (IL-1β), interleukin-2 (IL-2), interleukin-6 (IL-6), interleukin-23 (IL-23), granulocyte-macrophage colony stimulating factor (GM-CSF), eotaxin, tumor necrosis factor α (TNF-α) and tumor necrosis factor β (TNF-β) were increased in the blood plasma of SAMP8 mice and PrP-hAβPPswe/PS1^ΔE9^ mice, regulated upon activation normal T cell expressed and secreted factor (RANTES) and interferon-γ (INF-γ) increased and monocyte chemotactic protein-1 (MCP-1) decreased only in SAMP8 mice, while interleukin-17 (IL-17) increased only in PrP-hAβPPswe/PS1^ΔE9^ mice. Comparing with SAMR1 mice and C57 mice respectively, anti-inflammatory factor interleukin-4 (IL-4) were decreased in the blood plasma of SAMP8 mice and PrP-hAβPPswe/PS1^ΔE9^ mice, interleukin-5 (IL-5) and interleukin-10 (IL-10) decreased only in SAMP8 mice, while granulocyte colony stimulating factor (G-CSF) decreased only in PrP-hAβPPswe/PS1^ΔE9^ mice. *P < 0.05, **P < 0.01, ***P < 0.001, compared with control mice. Data represent mean ± S.D., n=10~15, Student T-test.

To investigate the state of immune system in SAMP8 mice and APP/PS1 mice, Luminex^®^ X-MAP^®^ technology was employed. Results showed that comparing with SAMR1 mice and C57 mice respectively, Interleukin (IL)-1β, IL-2, IL-6, IL-23, granulocyte-macrophage colony stimulating factor (GM-CSF), tumor necrosis factor(TNF)-α, TNF-β and chemotactic factor eotaxin were increased in the blood plasma of SAMP8 mice and APP/PS1 mice, interferon-γ (IFN-γ) and chemotactic factor regulated upon activation normal T cell expressed and secreted factor (RANTES) increased and chemotactic factor monocyte chemotactic protein-1 (MCP-1) decreased only in SAMP8 mice, while IL-17 increased only in APP/PS1 mice. Comparing with SAMR1 mice and C57 mice respectively, anti-inflammatory factor IL-4 were decreased in the blood plasma of SAMP8 mice and APP/PS1 mice, IL-5 and IL-10 decreased only in SAMP8 mice, while granulocyte colony stimulating factor (G-CSF) decreased only in APP/PS1 mice (Table 1). These data indicated that immune was aberrant in SAMP8 mice and APP/PS1 mice.

### The specific phenotype of cognition, neuroendocrine, immune and neuroendocrine immunomodulation molecular network in SAMP8 mice and APP/PS1 mice

SAMP8 mice as spontaneous model have been established through phenotypic selection from a common genetic pool of AKR/J, while APP/PS1 mice as gene-modified model were transgenic mice overproducing human APP and associated secretase. In order to distinguish them from cognitive, neuroendocrine and immune phenotype, principal component analysis (PCA) was performed in this study. The specific phenotype of neuroendocrine immunomodulation molecular network was delineated using MetaCore database and Cytoscape tools.

**Figure 2:**
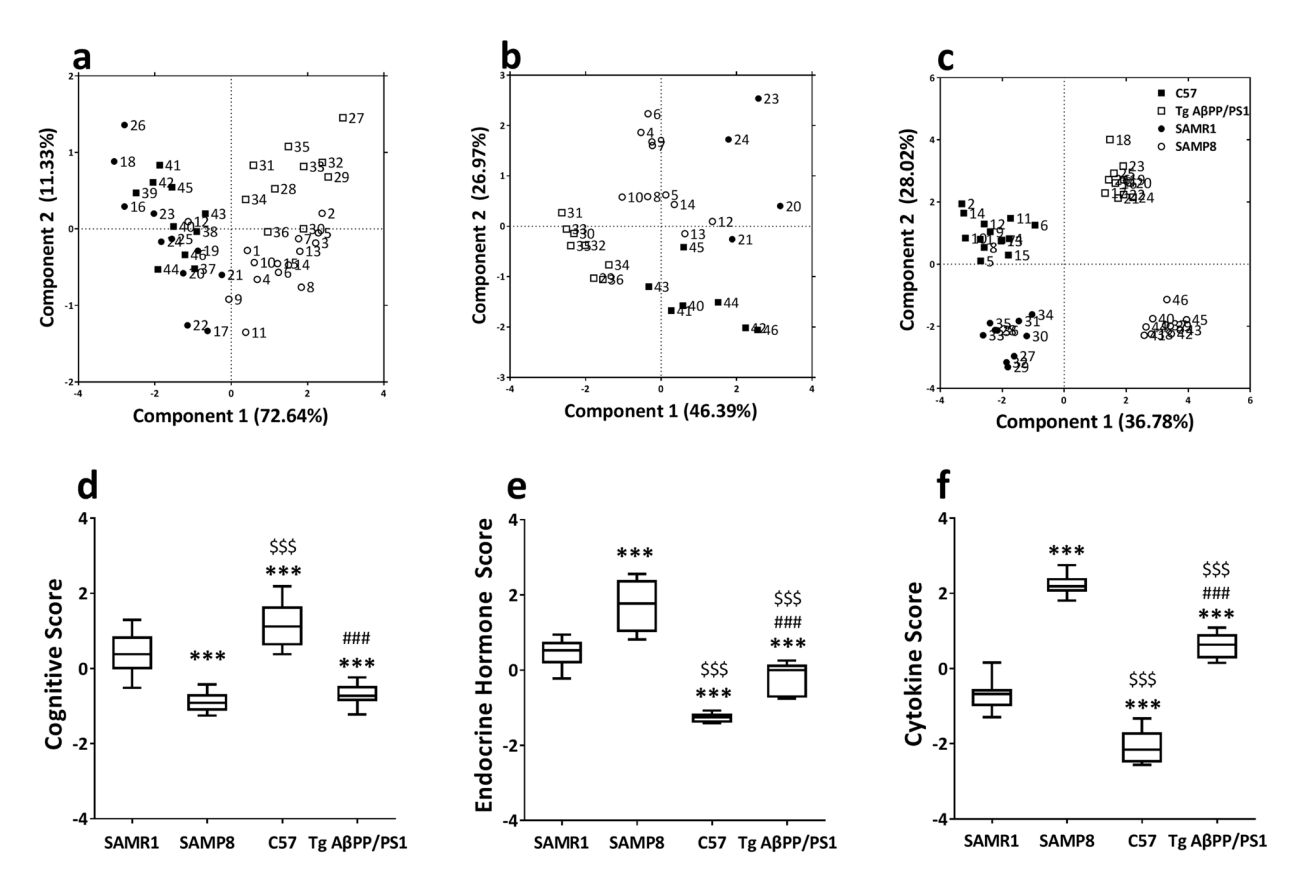
Principal component analysis of SAMP8 mice and PrP-hAβPPswe/PS1^ΔE9^ mice based on cognitive, neuroendocrine and immune phenotype Each axis was derived by principal component analysis (x: Component 1 and y: Component 2). Each point represents one of SAMR1 mice, SAMP8 mice, C57 mice and PrP-hAβPPswe/PS1^ΔE9^ mice. PCA based on cognitive performance of SAMR1 mice, SAMP8 mice, C57 mice and PrP-hAβPPswe/PS1^ΔE9^ mice **a.** Component 1(variance explained: 72.64%) contained spatial learning and memory ability (0.51) and object recognition memory (0.50), component 2 (variance explained: 11.33%) contained passive avoidance response (0.54) and active avoidance response (0.64), considered significant variance with a load below or equal to 0.50 (absolute value). The scores plot showed the different strains were approximately scattered into two different regions (SAMR1 mice and C57 mice at right region, SAMP8 mice and PrP-hAβPPswe/PS1^ΔE9^ mice at left region). PCA based on endocrine factor of SAMR1 mice, SAMP8 mice, C57 mice and PrP-hAβPPswe/PS1^ΔE9^ mice **b.** Component 1(variance explained: 46.39%) contained FSH (0.52), LH (0.49), corticosterone (0.41), component 2 (variance explained: 26.97%) contained ACTH (0.66), GnRH (0.54), considered significant variance with a load below or equal to 0.40 (absolute value). The scores plot showed the different strains were approximately scattered into four different regions (SAMR1 mice at bottom-left region, C57 mice at top-left region, SAMP8 mice at bottom-right region and PrP-hAβPPswe/PS1^ΔE9^ mice at top-right region). PCA based on cytokine of SAMR1 mice, SAMP8 mice, C57 mice and PrP-hAβPPswe/PS1^ΔE9^ mice **c.** Component 1(variance explained: 36.78%) contained IL-1β (0.39), IL-2 (0.36), TNF-α (0.32), TNF-β (0.36), component 2 (variance explained: 28.01%) contained IL-6 (0.44), GM-CSF (0.40), MCP-1 (0.33), IL-23 (0.32), INF-γ (−0.38), and MIP-1β (−0.39), considered significant variance with a load below or equal to 0.30 (absolute value). The scores plot showed the different strains were approximately scattered into four different regions (SAMR1 mice at top-right quadrant, C57 mice at bottom-right quadrant, SAMP8 mice at top-left quadrant and PrP-hAβPPswe/PS1^ΔE9^ mice at bottom-left quadrant). *n* = 46, principal component analysis by SAS 9.2 statistics package, the significance level was set at *P* < 0.05. The average scores of cognitive performance **d.**, endocrine hormone **e.**, and cytokine **f.** of SAMR1, SAMP8 mice, C57 mice and PrP-hAβPPswe/PS1^ΔE9^ mice in the principal component analysis. ^***^P < 0.001 compared with SAMR1 mice, ^###^*P* < 0.001 compared with C57 mice, ^$$$^*P* < 0.001 compared with SAMP8 mice. Data represent box and whiskers, Min to Max, *n* = 10~15, unpaired Student *T*-test.

PCA based on cognitive performance of SAMR1 mice, SAMP8 mice, C57 mice and APP/PS1 mice showed PC1 contained spatial learning and memory ability and object recognition memory, and PC2 contained passive avoidance response and active avoidance response classified SAMR1 mice and C57 mice to right region, SAMP8 mice and APP/PS1 mice to left region (Figure 2a) (supplemental material 1). Furthermore, the cognitive score in PCA distinguish SAMP8, C57 and APP/PS1 mice from SAMR1 mice, from APP/PS1 mice and C57 mice, C57 mice from SAMP8 mice (Figure 2d) (supplemental material 1).

PCA based on endocrine hormone of SAMR1 mice, SAMP8 mice, C57 mice and APP/PS1 mice showed that PC1 contained FSH, LH and CORT, and PC2 contained ACTH and GnRH scattered different strains into four different regions. SAMR1 mice were at bottom-left region, C57 mice at top-left region, SAMP8 mice at bottom-right region and APP/PS1 mice at top-right region (Figure 2b) (supplemental material 1). The endocrine hormone score in PCA distinguish SAMP8, C57 and APP/PS1 mice from SAMR1 mice, APP/PS1 mice from C57 mice, C57 from SAMP8 mice (Figure 2e) (supplemental material 1). It was important for this endocrine hormone score to differ SAMP8 from APP/PS1 mice (Figure 2e) (supplemental material 1).

PCA based on cytokine of SAMR1 mice, SAMP8 mice, C57 mice and APP/PS1 mice showed that PC1 contained IL-1β, IL-2, TNF-α and TNF-β, and PC2 contained IL-6, GM-CSF, MCP-1, IL-23, IFN-γ, and macrophage inflammatory protein-1β (MIP-1β) classified different strains into four different regions. SAMR1 mice were at top-right quadrant, C57 mice at bottom-right quadrant, SAMP8 mice at top-left quadrant and APP/PS1 mice at bottom-left quadrant (Figure 2c) (supplemental material 1). Furthermore, the cytokine score in PCA distinguish SAMP8, C57 and APP/PS1 mice from SAMR1 mice, SAMP8 and APP/PS1 mice from C57 mice (Figure 2f) (supplemental material 1). It was important for this cytokine score to differ SAMP8 from APP/PS1 mice (Figure 2f) (supplemental material 1).

**Figure 3:**
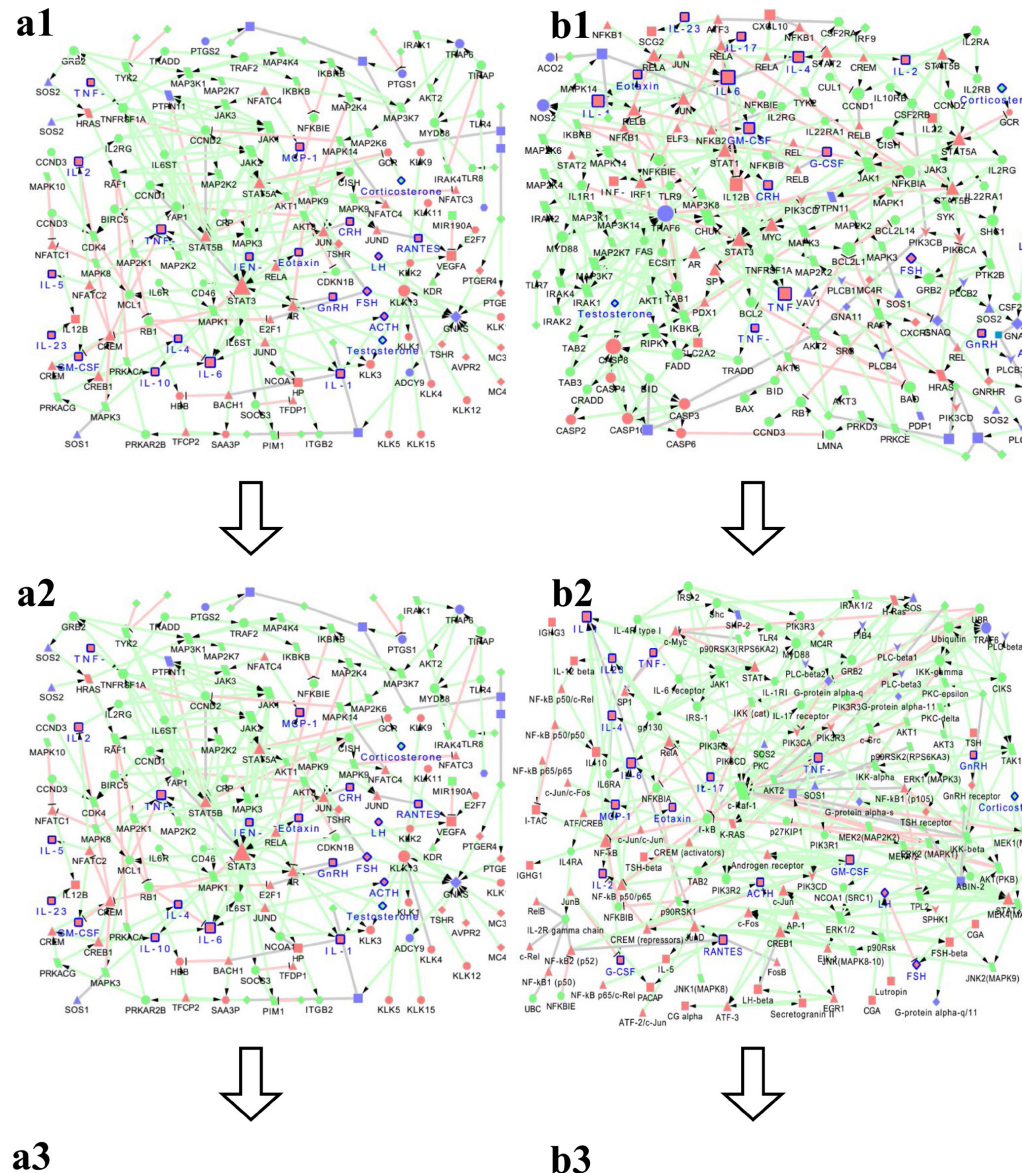

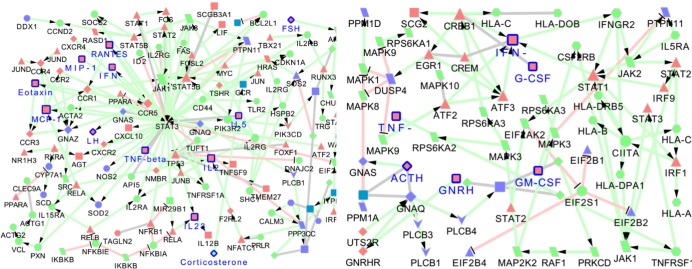
The MetaCore network of neuroendocrine immunomodulation in SAMP8 mice (a1) and PrP-hAβPPswe/PS1^ΔE9^ mice (b1), endocrine factors and cytokines correlated with cognitive impairments of SAMP8 mice and PrP-hAβPPswe/PS1^ΔE9^ mice analyzed by Pearson analysis (a2, b2) and multiple liner regression analyses (a3, b3) The differentially expressed molecules in SAMP8 mice (CRH, ACTH, CORT, GnRH, FSH, LH, T, IL-1β, IL-2, IL-6, IL-23, GM-CSF, INF-γ, TNF-α, TNF-β, RANTES, eotaxin, MCP-1, IL-4, IL-5, IL-10, total 21 seeds) and PrP-hAβPPswe/PS1^ΔE9^ mice(CRH, ACTH, CORT, GnRH, FSH, LH, T, IL-1β, IL-2, IL-6, IL-23, IL-17, GM-CSF, TNF-α, TNF-β, eotaxin, IL-4, G-CSF, total 18 seeds), the molecules correlated with cognitive impairments of SAMP8 mice (CRH, ACTH, CORT, GnRH, FSH, LH, T, IL-1β, IL-2, IL-6, IL-23, GM-CSF, INF-γ, TNF-α, TNF-β, RANTES, eotaxin, MCP-1, IL-4, IL-5, IL-10, total 21 seeds) and PrP-hAβPPswe/PS1^ΔE9^ mice(ACTH, CORT, GnRH, FSH, LH, IL-1β, IL-2, IL-6, IL-23, IL-17, GM-CSF, INF-γ, TNF-α, TNF-β, eotaxin, MIP-1β, IL-4, G-CSF, total 18 seeds) analyzed by Pearson analysis, the molecules correlated with cognitive impairments of SAMP8 mice (CORT, FSH, LH, IL-2, IL-23, TNF-β, RANTES, eotaxin, MCP-1, RANTES, MIP-1β, IL-5 total 12 seeds) and PrP-hAβPPswe/PS1^ΔE9^ mice(ACTH, GnRH, GM-CSF, INF-γ, TNF-β, G-CSF, total 6 seeds) analyzed by multiple liner regression analyses in the present study were submitted to MetaCore (https://portal.genego.com). And obtained a comparatively complete molecular interaction network for SAMP8 mice and PrP-hAβPPswe/PS1^ΔE9^ mice by the visualization tool of Cytoscape. Nodes marked with blue border and label are differentially expressed molecules in SAMP8 mice and PrP-hAβPPswe/PS1^ΔE9^ mice we measured. 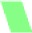 represent protein kinase, 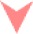 represent lipid kinase, 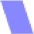 represent protein phosphatase, 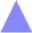 represent regulators (GDI, GAP, GEF), 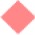 represent GPCR, 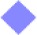 represent G-alpha, 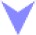 represent generic phospholipase, 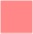 represent receptor ligand, 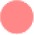 represent generic protease, 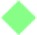 represent compound, 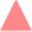 represent transcription factor, 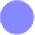 represent generic enzyme, 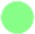 represent generic binding protein, 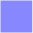 represent reaction, 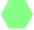 represent generic receptor. The size of the node corresponds to the number of its indegree. The green line with black arrow indicates activation. The red line with black small line segments indicates inhibition. The gray line indicates there is undefined interaction between nodes.

In order to obtain the specific phenotype of neuroendocrine immunomodulation (NIM) molecular network, network was constructed by submitting differential proteins in SAMP8 and APP/PS1 mice to MetaCore database using Cytoscape tools. Results showed that the NIM molecular network of SAMP8 mice contained 160 nodes and 261 edges with characteristic path length 5.436 (Figure 3a1) (supplemental material 2), and network of APP/PS1 mice contained 197 nodes and 335 edges with characteristic path length 4.776 (Figure 3b1) (supplemental material 2). The remarkable GO process and KEGG pathway of SAMP8 mice were “signal transduction” and “reproduction GnRH signaling”, and those of APP/PS1 mice were “defense response” and “Immune response TLR5, TLR7, TLR8 and TLR9 signaling pathways”(supplemental material 3).

### The endocrine hormone and cytokine correlated with cognition in SAMP8 mice and APP/PS1 mice

In order to find endocrine hormone and cytokine contributing to cognitive impairment in SAMP8 mice and APP/PS1 mice, Pearson correlation analysis was performed. Results showed the object recognition memory, spatial learning and memory ability, active and passive avoidance response were significantly correlated with most endocrine hormones and cytokines in SAMP8 and APP/PS1 mice (Table 2).

**Table 2:** Correlation between endocrine hormone/cytokines and cognitive performance of SAMP8 mice and PrP-hAβPPswe/PS1^ΔE^^9^ mice

Endocrine hormone/cytokines	SAMP8 mice								PrP–hAβPPswe/PS1^ΔE9^ mice							
	Object recognitionmemory		Spatial learning and memoryability		Passive avoidanceresponse		Active avoidanceresponse		Object recognitionmemory		Spatial learning and memoryability		Passive avoidanceresponse		Active avoidanceresponse	
	*R* value	*P* value	*R* value	*P* value	*R* value	*P* value	*R* value	*P* value	*R* value	*P* value	*R* value	*P* value	*R* value	*P* value	*R* value	*P* value
*HPA Axis*																
CRH	−0.63	0.01	uncorrelated		0.69	<0.01	−0.64	<0.01	uncorrelated		uncorrelated		uncorrelated		uncorrelated	
ACTH	uncorrelated		uncorrelated		uncorrelated		−0.46	0.05	uncorrelated		−0.40	0.04	0.48	0.01	uncorrelated	
CORT	−0.75	<0.01	−0.61	0.01	0.54	0.03	−0.65	0.01	−0.83	<0.01	uncorrelated		uncorrelated		−0.51	0.02
*HPG Axis*																
GnRH	−0.66	<0.01	−0.62	<0.01	uncorrelated		−0.61	<0.01	−0.55	<0.01	uncorrelated		0.54	0.01	−0.48	0.01
FSH	−0.49	0.03	uncorrelated		uncorrelated		−0.56	0.01	−0.46	0.03	−0.54	0.01	0.55	<0.01	−0.66	<0.01
LH	−0.74	<0.01	−0.50	0.03	0.56	0.01	−0.84	<0.01	−0.49	0.01	−0.56	<0.01	0.53	0.01	−0.66	<0.01
T	0.77	<0.01	uncorrelated		−0.47	0.04	0.78	<0.01	uncorrelated		uncorrelated		uncorrelated		uncorrelated	
*Proinflammatory factor*																
Interleukin																
IL-1β	−0.60	<0.01	−0.62	<0.01	0.30	0.01	−0.63	<0.01	−0.57	<0.01	−0.34	<0.01	0.36	<0.01	−0.54	<0.01
IL-2	−0.56	<0.01	−0.51	<0.01	0.24	0.04	−0.65	<0.01	−0.31	<0.01	−0.28	<0.01	0.24	0.01	−0.42	<0.01
IL-6	−0.32	0.01	−0.42	<0.01	0.24	0.05	−0.52	<0.01	−0.38	<0.01	−0.30	<0.01	0.36	<0.01	−0.38	<0.01
IL-23	−0.58	<0.01	−0.60	<0.01	0.32	0.01	−0.54	<0.01	−0.37	<0.01	uncorrelated		0.25	0.01	−0.34	<0.01
IL-17	uncorrelated		uncorrelated		uncorrelated		uncorrelated		−0.20	0.04	−0.23	0.02	0.24	0.02	−0.33	<0.01
Colony stimulating factor																
GM-CSF	−0.46	<0.01	−0.47	<0.01	0.25	0.04	−0.49	<0.01	−0.29	<0.01	−0.27	0.01	0.37	<0.01	−0.42	<0.01
Interferon																
IFN-γ	−0.50	<0.01	−0.54	<0.01	0.26	0.03	−0.55	<0.01	0.32	<0.01	uncorrelated		−0.21	0.03	0.25	0.01
Tumor necrosis factor																
TNF-α	−0.54	<0.01	−0.50	<0.01	uncorrelated		−0.53	<0.01	−0.41	<0.01	−0.35	<0.01	0.34	<0.01	−0.51	<0.01
TNF-β	−0.46	<0.01	−0.54	<0.01	uncorrelated		−0.57	<0.01	−0.45	<0.01	−0.35	<0.01	0.45	<0.01	−0.56	<0.01
Chemotactic factor																
RANTES	−0.46	<0.01	uncorrelated		0.26	0.02	−0.35	<0.01	uncorrelated		uncorrelated		uncorrelated		uncorrelated	
eotaxin	−0.57	<0.01	−0.53	<0.01	0.31	0.01	−0.66	<0.01	−0.38	<0.01	−0.33	<0.01	0.37	<0.01	−0.49	<0.01
MCP-1	0.32	0.01	0.32	0.01	uncorrelated		0.43	<0.01	uncorrelated		uncorrelated		uncorrelated		uncorrelated	
MIP-1β	uncorrelated		uncorrelated		uncorrelated		uncorrelated		0.20	0.04	uncorrelated		uncorrelated		uncorrelated	
*Antiinflammatory factor*																
IL-4	0.39	<0.01	0.29	0.01	uncorrelated		0.40	<0.01	0.27	0.01	0.31	<0.01	−0.23	0.02	0.35	<0.01
IL-5	0.31	0.01	uncorrelated		uncorrelated		0.36	<0.01	uncorrelated		uncorrelated		uncorrelated		uncorrelated	
IL-10	uncorrelated		0.28	0.02	uncorrelated		0.34	< 0.01	no data		no data		no data		no data	
G-CSF	uncorrelated		uncorrelated		uncorrelated		uncorrelated		0.30	<0.01	0.35	<0.01	−0.21	0.04	0.40	<0.01

The object recognition memory (preferential index in novel object recognition test) was significantly correlated with CRH, CORT, GnRH, FSH, LH, T, IL-1β, IL-2, IL-5, IL-6, GM-CSF, INF-γ, TNF-α, TNF-β, MCP-1, RANTES, eotaxin, IL-4 and IL- 23 in SAMP8 mice, while CORT, GnRH, FSH, LH, IL-1β, IL-2, IL-6, IL-23, IL-17, GM-CSF, INF-γ, TNF-α, TNF-β, MIP-1β, eotaxin, IL-4 and G-CSF in PrP-hAβPPswe/PS1^ΔE9^ mice. The spatial learning and memory ability (number of crossing plate in Morris water maze test) of SAMP8 mice was significantly correlated with CORT, GnRH, LH, IL-1β, IL-2, IL-6, GM-CSF, INF-γ, MCP-1, TNF-α, eotaxin, IL-4, IL-23, IL-10 and TNF-β. The variance correlated with spatial learning and memory ability was ACTH, FSH, LH, IL-1β, IL-2, IL-6, IL-17, GM-CSF, TNF-α, TNF-β, eotaxin, IL-4 and G-CSF in PrP-hAβPPswe/PS1^ΔE9^ mice. The passive avoidance response (number of errors in step down test) of SAMP8 mice was correlated with CRH, CORT, LH, T, IL-1β, IL-2, GM-CSF, INF-γ, TNF-α, RANTES, eotaxin, IL-23, TNF-β, and that of PrP-hAβPPswe/PS1^ΔE9^ mice was correlated with ACTH, GnRH, FSH, LH, IL-1β, IL-2, IL-6, IL-17, GM-CSF, INF-γ, TNF-α, TNF-β, eotaxin, IL-4, G-CSF, and IL-23. The active avoidance response (successful avoidance times in shuttle-box test) of SAMP8 mice was correlated with CRH, ACTH, CORT, GnRH, FSH, LH, T, IL-1β, IL-2, IL-5, IL-6, GM-CSF, INF-γ, TNF-α, MCP-1, RANTES, eotaxin, IL-4, IL-23, TNF-β and IL-10, that of PrP-hAβPPswe/PS1^ΔE9^mice was correlated with CORT, GnRH, FSH, LH, IL-1β, IL-2, IL-6, GM-CSF, INF-γ, TNF-α, eotaxin, IL-4, G-CSF, IL-23, TNF-β. n=20~26, two-tailed Pearson analysis with 95% confidence interval by Graphpad Prism 6.0, the correlation was considered statistically significant if *P* < 0.05.

In order to obtain the NIM molecular network correlated with cognitive impairment, we submitted the factors significantly correlated with cognition of SAMP8 mice and APP/PS1 mice, obtained by Pearson correlation analysis, to MetaCore database, respectively. Results showed that the NIM molecular network correlated with cognitive impairment of SAMP8 mice contained 160 nodes and 261 edges with characteristic path length 5.436 (Figure 3a2), and network of APP/PS1 mice contained 161 nodes and 293 edges with characteristic path length 4.549 (Figure 3b2) (supplemental material 2). The remarkable GO process and KEGG pathway of SAMP8 mice were “signal transduction” and “Reproduction GnRH signaling”, and those of APP/PS1 mice were “signal transduction” and “Immune response Gastrin in inflammatory response”(supplemental material 3).

### The neuroendocrine immunomodulation network underlying cognitive impairment in SAMP8 mice and APP/PS1 mice

Based on PCA results, in order to clarify the underpinnings of learning and memory deficits by zooming out to the molecular level to search for the key causal molecules in many contributions of NIM network. Stepwise multiple linear regression analysis was performed among all the endocrine hormone and cytokine. Results showed that CORT, IL-5, MCP-1, MIP-1β, TNF-β and IL-23 account for 96% of passive avoidance response in SAMP8, while GM-CSF for 46% in APP/PS1 mice (Table 3). LH and TNF-β account for 88% active avoidance response in SAMP8, and TNF-β for 76% in APP/PS1 mice (Table 3). FSH, LH, IL-2, MCP-1, RANTES and IL-23 account for 95% spatial learning and memory ability in SAMP8, and ACTH and G-CSF for 58% in APP/PS1 mice (Table 3). GM-CSF, RANTES, eotaxin and TNF-β account for 98% object recognition memory in SAMP8, and ACTH, GnRH, IFN-γ and TNF-β for 89% in APP/PS1 mice (Table 3).

**Table 3:** Multiple linear regression model of neuroendocrine immunomodulation underlying cognitive impairment in SAMP8 mice and PrP-hAβPPswe/PS1^ΔE^^9^ mice

Predictor	SAMP8 mice							PrP-hAβPPswe/PS1^ΔE9^ mice							
	Coefficient	*t* value	*P* value	Model summary				Predictor	Coefficient	*t* value	*P* value	Model summary			
				*F* value	*P* value	*R^2^*	Adj *R^2^*					*F* value	*P* value	*R^2^*	Adj *R^2^*
*Passive avoidance response*				39.24	<0.01	0.96	0.94					22.20	<0.01	0.46	0.43
Intercept	4.26	2.12						Intercept	−2.77	−2.00					
CORT	<0.01	2.61	0.03					GM-CSF	0.20	4.49	<0.01				
IL-5	0.08	3.84	<0.01												
MCP-1	−0.03	−2.89	0.02												
MIP-1β	−0.13	−2.75	0.02												
TNF-β	−0.09	−6.63	<0.01												
IL-23	0.15	11.55	<0.01												
*Active avoidance response*				64.27	<0.01	0.88	0.87					30.35	<0.01	0.76	0.75
Intercept	34.78	15.34						Intercept	19.73	18.06					
LH	−7.74	−3.22	0.01					TNF-β	−0.09	−8.81	<0.01				
TNF-β	−0.15	−5.12	<0.01												
*Spatial learning and memory ability*				38.25	<0.01	0.95	0.92					11.61	<0.01	0.58	0.53
Intercept	9.78	5.89						Intercept	0.17	0.07					
FSH	9.55	4.22	<0.01					ACTH	−0.03	−2.43	0.03				
LH	−2.52	−2.76	0.02					G-CSF	0.07	2.74	0.01				
IL-2	−0.07	−2.92	0.01												
MCP-1	−0.04	−3.89	<0.01												
RANTES	0.10	3.57	<0.01												
IL-23	−0.07	−3.50	<0.01												
*Object recognition memory*				146.22	<0.01	0.98	0.97					30.60	<0.01	0.89	0.86
Intercept	151.62	27.92						Intercept	102.47	6.84					
GM-CSF	−0.92	−2.83	0.01					ACTH	−0.24	−4.03	<0.01				
RANTES	0.32	2.32	0.03					GnRH	−7.22	−3.43	<0.01				
eotaxin	−0.13	−4.15	<0.01					INF-γ	2.03	3.18	0.01				
TNF-β	−0.30	−4.47	<0.01					TNF-β	−0.17	−2.65	0.02				

The independent variable (IL-1β, IL-2, IL-5, IL-6, IL-17, GM-CSF, INF-γ, TNF-α, MCP-1, MIP-1β, RANTES, eotaxin, IL-4, G-CSF, IL-23, TNF-β, ATCH, FSH, LH, GnRH, CRH, T and CORT) contributes to the object recognition memory, spatial learning and memory, passive avoidance response and active avoidance response respectively in SAMP8 mice and PrPhAβPPswe/PS1^ΔE9^ mice by stepwise multiple regression analyses. The coefficient is the estimate of regression coefficients, t value is test statistic for this test which is based on the t distribution, P-value is the occurrence probability of the test statistic values that are either equal to the one obtained from the sample or more unfavorable to hypothesis than the one obtained from the sample, F value (associated with P-value that is less than 0.01) indicates a significant relationship between the dependent variable and at least one of independent variables, R2 indicates how well the regression model fits the observed data. Adj R2 is R2 being adjusted in statistic analysis. Each P-value is less than 0.05. n=20~26, statistical analyses were performed using the SAS 9.2 statistics package, the significance level was set at P < 0.05.

For endocrine hormone contributing to cognitive impairment, CORT, LH and FSH were important in SAMP8, while ATCH and GnRH in APP/PS1 mice (Table 3). This indicated abnormal HPA and HPG axis trigger in part the cognitive deficit in these two animal AD models. For cytokines contributing to cognitive impairment, chemotactic factor MCP-1, MIP-1β, RANTES and eotaxin only decide deterioration of cognitive performance in SAMP8, not APP/PS1 mice (Table 3). Pro-inflammatory factor IL-23 contributed to cognitive impairment in SAMP8, while colony stimulating factor G-CSF in APP/PS1 mice (Table 3). The imbalance between Th1 and Th2 cell contributed to cognitive impairment in SAMP8 mice and APP/PS1 mice. But Th1 cell acting as cell immunity accounted for learning and memory deficiencies in SAMP8 mice(Supplemental material 5a), while Th2 cell acting as humoral immunity in APP/PS1 mice (Supplemental material 5b).

In order to obtain the NIM network underlying cognitive impairment, we submitted the factors significantly correlated with cognition of SAMP8 mice and APP/PS1 mice, obtained by multiple linear regression analysis, to MetaCore database, respectively. Results showed that the NIM network underlying cognitive impairment of SAMP8 mice contained 127 nodes and 178 edges with characteristic path length 3.964 (Figure 3a3), and network of APP/PS1 mice contained 67 nodes and 84 edges with characteristic path length 6.533 (Figure 3b3) (supplemental material 2). The remarkable GO process and KEGG pathway of SAMP8 mice were “response to organic substance” and “Immune response IL-2 activation and signaling pathway”, and those of APP/PS1 mice were “cell surface receptor signaling pathway” and “Immune response antiviral actions of interferons” (supplemental material 3).

**Figure 4:**
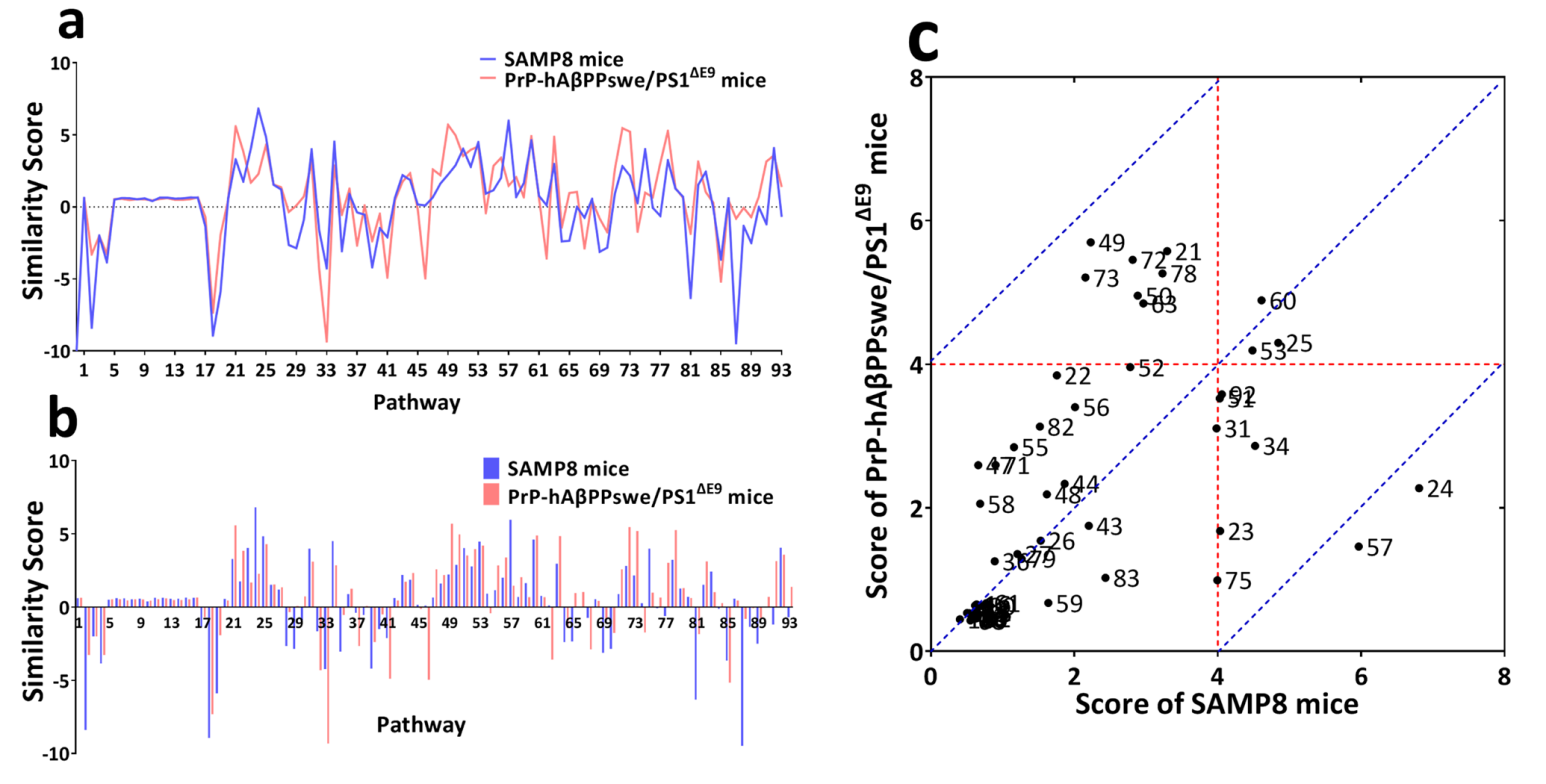
Network fingerprint analysis NIM network underlying cognitive impairment of SAMP8 and PrP-hAβPPswe/PS1^ΔE9^ mice MetaCore expanded networks were processed by network fingerprint frameworks as described previously. *SSscore* on each of 93 KEGG pathways **a.** and **b.** indicated that the set of pathways contributed to learning and memory deficits in SAMP8 mice (SAD) and PrP-hAβPPswe/PS1^ΔE9^ mice (FAD) were basically similar, however, the *SSscore* values were different in term of certain interesting pathways. Two-dimensional representation of *SSscore* **c.** This scatterplots represent the contribution of each pathway to learning and memory deficits of AD mice, X axis was the corresponding *SSscore* of SAMP8 mice on all pathways, Y axis was *SSscore* of PrP-hAβPPswe/PS1^ΔE9^ mice on all pathways. Arbitrarily, any pathway with *SSscore* >4 was considered to be a specific functional pathway contributed to cognition deficits. The scores plot showed the common pathways related with cognition impairments of SAMP8 and PrP-hAβPPswe/PS1^ΔE9^ mice (near the red dotted line) were Chemokine signaling pathway (#60) and T cell receptor signaling pathway (#53), the different pathways (near the blue dotted line) were Cytokine-cytokine receptor interaction (#24) and Natural killer cell mediated cytotoxicity (#57) that specific pathways in MetaCore network of SAMP8 mice. These results indicated that the Chemokine signaling pathway and T cell receptor signaling pathway might be potential target pathways for future AD drug development. Regarding to the SAD, the Cytokine-cytokine receptor interaction and Natural killer cell mediated cytotoxicity should attract considerable attention as specific drug target pathways, beyond that two common pathways.

To identify the differences and similarities between NIM network underlying cognitive impairment of SAMP8 and APP/PS1 mice, we adapted the “network fingerprint” frameworks introduced by our colleagues recently [19]. The MetaCore networks in Figure 3a3 and Figure 3b3 were feed into this computational framework and making systematic comparisons to well-characterized KEGG pathways. The output standardized similarity score (*SSscore_i_*) were graphically displayed in Figure 4a and Figure 4b (Supplemental material 4). Since only positive values are meaningful for functional association purpose, we only plotted positive values as Figure 4c. Arbitrarily, any pathway with *SSscore* >4 was considered to be a specific functional pathway contributed to cognition deficits. The scores plot showed the common pathways correlated with cognition impairments of SAMP8 and APP/PS1 mice (near the red dotted line) were chemokine signaling pathway (#60) and T cell receptor signaling pathway (#53) (Figure 4c), the different pathways (near the blue dotted line) were cytokine-cytokine receptor interaction (#24) and natural killer cell mediated cytotoxicity (#57) and these were specific pathways in MetaCore network of SAMP8 mice (Figure 4c).

## DISCUSSION

The disturbances of HPA axis contributed to the cognitive impairments and psychological symptoms which occurred in AD and thus participated in the etiology [20, 21]. Epidemiological and biochemical studies indicated a relation between hormones of the HPG axis and cognitive decline [2, 22–24]. Our previous study had shown that impaired cognitive function of SAMP8 was related to the age-related decrease in serum testosterone [25]. SAMP8 mice aged 26-31 weeks exhibited shortened estrous cycles and high FSH levels compared to SAMP8 mice aged 8-12 weeks [26]. And there was an age-related increase LH concentration, which higher in SAMP8 mice than SAMR1 mice, after 7 months [27]. Our these previous studies indicated that the HPG axis of SAMP8 mice was ageing-induced malfunction, as well as the hyperactive HPA axis of SAMP8 mice which might induce the neurodegeneration [28–31]. The present study indicated that SAMP8 mice at the age of 9 months exhibited the imbalance of HPA and HPG axis. However, there was less study on the HPA axis and HPG axis of APP/PS1 mice and analogous models (Supplemental material 6). We firstly detected the changes of HPA axis and HPG axis of APP/PS1 mice, and the correlations between cognitive impairment and them. According to our results, both HPA axis and HPG axis in APP/PS1 mice were all malfunction.

Studies shows cytokines play a critical role in pro- and anti-inflammatory processes in AD, and complex cognitive processes [32]. Compared the health, the levels of IL-1, IL-4, IL-6, IL-10, IL-12, IL-16, IL-18, TNF, TGF-β, MCP and IL-8 are reportedly disordered in AD [33]. This unbalanced state of cytokines have been associated with cognitive decline and dementia [34, 35]. In this study, indiscriminate cytokine secretion was one of potential mechanism underlying cognitive impairments in APP/PS1 mice and SAMP8 mice, although the direct reason causing AD-like behaviors and pathology of APP/PS1 mice and SAMP8 mice was considered the overexpression of the hAPP encoding gene with Swedish mutation together with the mutant PS1 gene and accelerated aging respectively.

**Figure 5:**
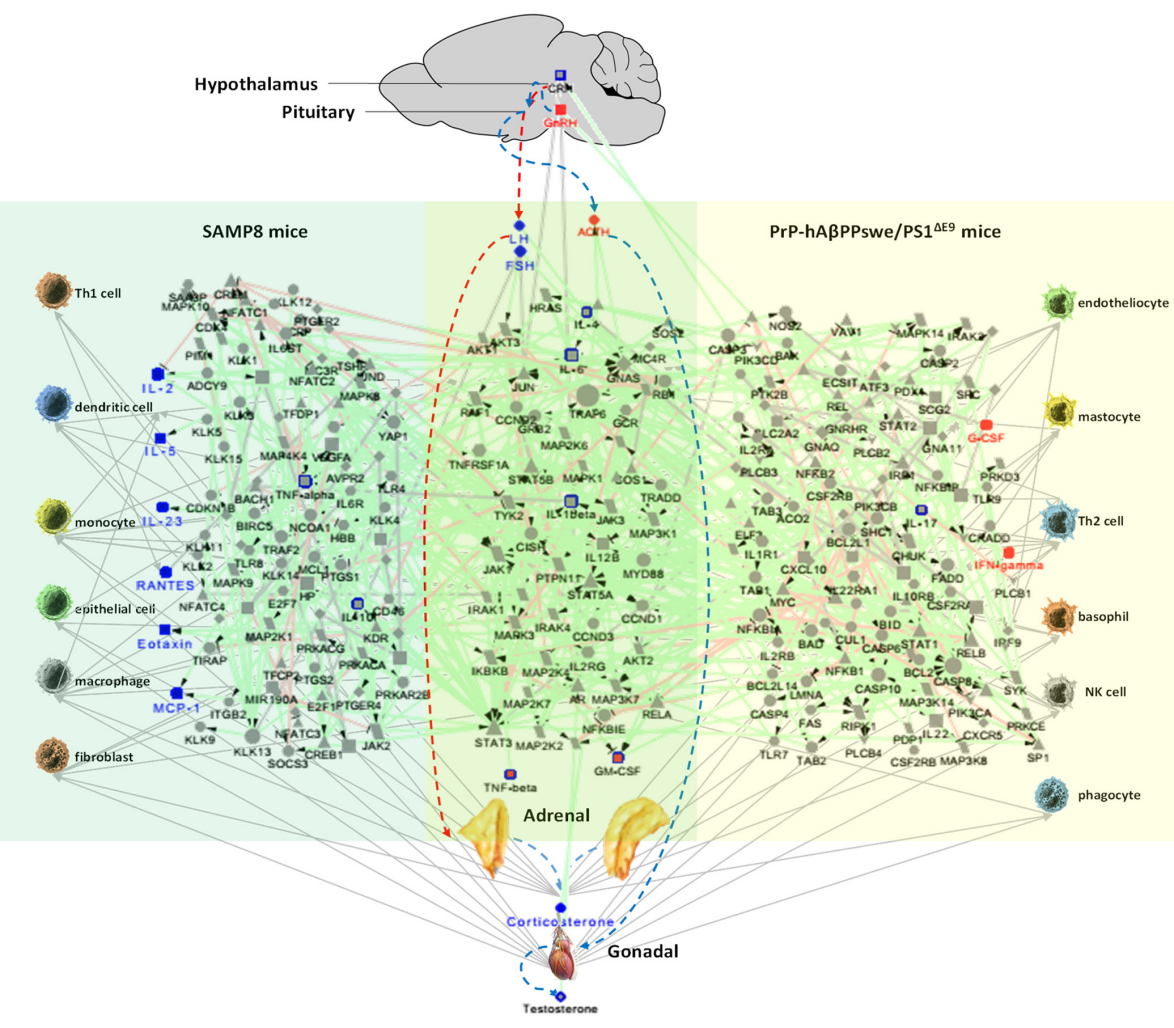
The neuroendocrine immunomodulation network underlying cognitive impairment in in SAMP8 and PrP-hAβPPswe/PS1^ΔE9^ mice The red dotted line indicates HPA axis, and blue dotted line indicates HPG axis. Blue nodes are the molecules correlated with the cognitive function of SAMP8 mice, red nodes are the molecules correlated with the cognitive function of PrP-hAβPPswe/PS1^ΔE9^ mice, red nodes marked with blue border are the common molecules correlated with the cognitive function of SAMP8 and PrP-hAβPPswe/PS1^ΔE9^ mice, gray nodes with blue border are the other differentially expressed molecules in SAMP8 mice and PrP-hAβPPswe/PS1^ΔE9^ mice we measured. The light blue region is exclusive network for SAMP8 mice, the light green region is common network between SAMP8 mice and PrP-hAβPPswe/PS1^ΔE9^ mice, and the light yellow region is exclusive network for PrP-hAβPPswe/PS1^ΔE9^ mice. The green line with black arrow indicates activation. The red line with black small line segments indicates inhibition. The gray line indicates there is undefined interaction between nodes. The gray line with gray arrow indicates there is an interaction between nodes.

In the present study, we firstly showed CORT, LH and FSH correlated with the cognitive function of SAMP8 mice, ACTH and GnRH correlated with that of APP/PS1 mice. Moreover, TNF-β and GM-CSF correlated with the cognitive function of SAMP8 and APP/PS1 mice, while IL-2, IL-5, IL-23, MCP-1, MIP-1β, RANTES, and eotaxin only correlated with the cognitive function of SAMP8 mice, and IFN-γ, G-CSF only correlated with that of APP/PS1 mice (Figure 5). In a clinical study, testosterone and gonadotropins had been correlated with cognitive impairment in normal male and the modulation of Aβ metabolism [36]. In another clinical cross-sectional study, a positive correlation between LH and TNF-α had been described in people with AD [37]. In mice, reduction of glucocorticoid receptor signaling stimulated the HPA axis and impacted cognition [38, 39], and deletion of glucocorticoid receptor could elevate corticosterone concentration and aggravate cognitive impairment [40, 41]. It is well known that the APP/PS1 mice as FAD animal model are induced by APP/PS1 transgene and SAMP8 mice as SAD animal model are induced by accelerated aging. However our present study indicated the abnormal NIM network contributed the impairment of cognition in both APP/PS1 transgene and SAMP8 mice. This is significant for further understanding the pathogenesis of AD and can provide useful information for research and development of AD therapeutic drug.

Comparing between the NIM network of APP/PS1 and SAMP8 mice, we concluded that there were two differences between them. The first is the T helper cell polarization in NIM network contributing to cognitive impairment. The Th1 cell acting as cell immunity accounted for learning and memory deficiencies in SAMP8 mice, while only Th2 cell acting as humoral immunity in APP/PS1 mice. It has been demonstrated that the immunization with Aβ peptide results in prevention of Aβ plaque formation and amelioration of established plaques in the brain of transgenic mouse models of AD [42, 43], and showed enhanced Th2 and down-regulated Th1 immunity [44–46]. This explains why Th2 cell acting as humoral immunity accounted for learning and memory deficiencies in APP/PS1 mice. There were many studies reported aging led to increased Th1 and Th2 cell numbers and a decreased Th2/Th1 ratio [47, 48], while SAMP8 mice show the classic characteristic of AD due to aging. These indicated that the overload of Aβ peptide caused the enhanced Th2 cell immunity in APP/PS1 mice induced by APP/PS1 transgene and Th1 cell in SAMP8 mice induced by aging. The second difference is the pathways in NIM network contributing to cognitive deterioration of both AD mouse model. The cytokine-cytokine receptor interaction (#24) and natural killer cell mediated cytotoxicity (#57) were the specific pathways in NIM network of SAMP8 mice (Figure 4c). NK cells represent a subpopulation of lymphocytes involving in innate immunity [49], as well as the regulation of the immune response through cytokine and chemokine production that activates other cellular components of innate and adaptive immunity [50]. It was found in AD patients that the significant negative correlations among the spontaneous release of cytokines from NK cells and the decrease of cognitive function [51, 52]. The dysfunction of NK cells substantially accelerated Aβ pathogenesis and also exacerbated the neuroinflammatory phenotype [53]. Alterations of NK cell cytotoxicity control and NK-derived cytokine release were involved in the neuroinflammatory mechanism related to neurodegeneration and dementia progression in AD [54]. These indicated that chronic neuroinflammation may be one cause inducing progressive neurodegeneration in SAMP8 mice. That the NIM network is different between SAMP8 and APP/PS1 mice can provide useful information relating to understanding the relationship between NIM network and AD. Therefore, when these two AD animal models were used in the research and development of AD drug, besides cognitive behavior and relative pathological feature, it is requisite to take into account the humoral immunity in APP/PS1 mice, cell immunity and neuroinflammation in SAMP8 mice.

On above all, the present study provided a new insight into the mechanisms underlying cognitive impairments in SAMP8 and APP/PS1 mice. The mechanisms of NIM network underlying cognitive impairment in AD animal model suggested the restoration of NIM network might be used to assess therapeutic approaches to AD.

## MATERIALS AND METHODS

### Experimental animals

SAMR1 and SAMP8 strains were obtained from our breeding colonies, maintained as inbred strains from original couples provided by Professor T. Takeda (Department of Senescence Biology, Kyoto University). There were male 9-month-old SAMR1 (*n* = 10) and SAMP8 (*n* = 10) mice were utilized in two experimental groups. The APP/PS1 mice were obtained from Beijing HFK Bioscience CO., LTD.. The 11 male 12-month-old heterozygous transgenic mice, which harboring an FAD-linked mutant AβPPswe/PS1^ΔE9^ co-expressing PS1 and a chimeric mouse-human AβPP 695 with mutations (K595N, M596L) that are linked to Swedish pedigrees *via* the mouse prion protein promoter and 15 age-matched wild type mice were used in two experimental groups. All animal experiments were performed in accordance with the National Institutes of Health Guide of the Care and Use of Laboratory Animals.

All behavioral tests were performed between 19:00 p.m. and 6:00 a.m. (Beijing time). Following the behavioral tests, the plasma, hypothalamus and pituitary was collected for cytokine and hormone measurements.

### Novel object recognition test

The procedure was according to Rick A bevins & Toyce Besheer (2006) [55]. The experimental procedure contains 3 phases: pre-training, training and testing. On the 1st and 2nd day, the mouse was allowed to acclimatize for 20 min per day. On the 3rd day, each mouse was allowed to explore the sample objects for 16 min. One hour later, the mouse was placed back to the similar chamber that one of the two identical objects was switched to a new one, to start a 4 min testing phase. On the 4th day (24 hours later), again the mouse was permitted to explore the diverse objects freely for 4 min. In the testing session, the preferential index (PI) was calculated using the following formula:

$$\text{preferential index}=\frac{\text{Time exploring novel object}}{\left(\text{Time exploring novel object}+\text{Time exploring identical object}\right)}\times 100\%$$

### Shuttle box test

The procedure of shuttle-box was according to Cheng, et al. (2011) [56]. Working memory was evaluated by the shuttle box apparatus (Med Associates Inc.). Training session began with an acclimatization to the chambers of 2 min followed by 30 trails, and inter-trial interval was 30 s. The tone (60dB) and light (8W) were used as the conditioned stimulus, for 10s. Followed by the unconditioned stimulus, an electrical foot shock (0.2 mA), for 5s. The shuttle-box procedure was performed for 5 consecutive days. At the 6th day, all mice were submitted to another session (no shock) of shuttle-box to test the level of the learning and memory ability. The number of active avoidances was recorded during the test.

### Step-down test

The procedure of step-down was according to He, et al. (2010), Shi, et al. (2010) and Luo, et al. (2012) [57–59]. On the 1st day, mouse was allowed to acclimatize for 2 min. The learning trail, if the mouse stepped down from the platform (error) with all four paws, they received an aversive foot electric shock (36V, AC), and the learning course was performed for 10 min. The number of errors and the time of the mice first stayed on the platform were scored. The testing trials (day 2-7), the procedure was repeated at the same time, testing time was 3 min. The number of errors was recorded.

### Morris water maze test

The procedure of Morris-water maze test was according to Vorhees CV and Williams MT (2006) [60]. The spatial learning phase consisted of 4 trials per day for 5 days, and one additional day (6th day) for probe trial. In the spatial learning phase, each mouse was placed on the platform for 60s before the first trial, then released into the water, allowed to find platform within 60s. If the mouse did not find the platform within 60s, it was gently led to the platform and allowed to remain there for 10s, the latency time was scored as 60s. The latency time was recorded as measures for spatial learning. For the spatial memory phase, the platform was removed, and the mouse was released into the water at a novel position and allowed to swim freely within 60s. The dependent measure for the spatial memory was the time in the target quadrant and the number of crossing the platform.

### Multiplex bead analysis

Cytokine were measured according to the manufacturer's instructions of multiplex bead analysis (Millipore Corp.). The samples were analyzed by Luminex 200^TM^ (Luminex). The concentrations of IL-1β, IL-2, IL-5, IL-17, IL-6, IL-4, IL-10, GM-CSF, G-CSF, IFN-γ, TNF-α, MCP-1, RANTES, eotaxin, MIP-1β were detected by a milliplex map kit (MCYTOMAG-70K), and IL-23, TNF-β were detected by another milliplex map kit (MGAMMAG-300K).

### Enzyme-linked immunosorbent assay

The level of plasma CORT in HPA axis of the mice were measured with precoated corticosterone ELISA kit (EC3001-1, ASSAYPRO) according to the manufacturer's instruction. The absorbance was measured at 450nm with a reference wavelength of 570 nm using Enspire ^TM^ multilabel reader 2300 (Perkin Elmer, Turku, Finland).

### Radioimmunoassay of hypothalamic and hypophyseal hormones

The hypothalamuses and pituitaries were weighed and boiled in 1 mL saline for 5 min. Peptides were extracted by homogenizing in 0.5 mL of 1 M glacial acetic acid followed by centrifuging the mixture at 3000 rpm for 30 min. CRH and ACTH of HPA axis, GnRH, LH, and FSH of HPG axis in the supernatants were determined with ^125^I-ATCH RIA kit (North Institute of Biological Technology), ^125^I-LH RIA kit (North Institute of Biological Technology), ^125^I-FSH RIA kit (North Institute of Biological Technology), ^125^I-CRH RIA kit (Department of Neurobiology of the Second Medical University ), ^125^I-GnRH RIA kit (Department of Neurobiology of the Second Medical University), respectively.

### Immunochemiluminescence assay

The level of plasma T in HPG axis of the mice was measured with Acces Immunoassay System (Beckman Coulter), access testosterone (33560, Beckman Coulter) and access testosterone calibrators (33565, Beckman Coulter). The entire measurement processed according to the scheduled program automatically.

### Principal component analysis

PCA is a classical multivariate technique, the aim is to extract the important information from numerous (*n*) possibly correlated variables (*M_1_*, *M_2_*, …, *M_n_*) and to represent it as a set of fewer variables, named principal components (PC) [61]. In the present study, a data matrix with *m* observations on *k_1_*, *k_2_* and *k_3_* variables (*m* = 46, the number of individuals in the entire data set, *k_1_* = 4, cognitive markers, *k_2_* = 7, neuroendocrine markers, *k_3_* = 16, immune markers). We chose the PC1 and PC2 to plot, in order to distinguish SAMR1 and SAMP8 mice, C57 and APP/PS1 mice from cognitive, neuroendocrine and immune phenotype. The PCA was processed SAS 9.2 (SAS Institute Inc.), and visualized by GraphPad Prism^®^, version 6.

### Multiple linear regression analysis

Multiple linear regressions analysis is a modelling technique for investigation of the relationship between one dependent variable and several independent variables. In the present study, in order to find the most important molecule respectively contributing to 4 types of cognitive impairments in SAMP8 mice and APP/PS1 mice, stepwise multiple linear regression analysis was performed. The dependent variables were the type of cognitive impairments with 46 observations, and the independent variables were 23 molecules of endocrine hormone and cytokine. Statistical analyses were performed SAS 9.2. The significance level was set at *P* < 0.05.

### Metacore network analysis

The procedure of Metacore network analysis was according to Cheng, et al. (2013) [62]. We submitted the differential molecules, the molecules correlated with cognition analyzed by Pearson correlation analysis, and the most important molecules contributing to cognitive impairment analyzed by multiple linear regression analysis in the present study to the MetaCore database, respectively. To obtain comparatively complete networks for SAMP8 and APP/PS1 mice, the “Auto expand” algorithm was employed, and visualized by Cytoscape, version 3.2.1 [63]. For the biological and functional annotation of the networks, Gene Ontology (GO) analysis and Kyoto Encyclopedia of Genes and Genomes (KEGG) pathway enrichment were performed based on the MetaCore database. The top ten GO processes and KEGG pathways (*P* < 10^−3^ and FDR < 0.01) were considered to be a specific function and biological pathway networks.

### Network fingerprint analysis

MetaCore expanded networks were processed by network fingerprint frameworks as described previously [19]. Each mice produced three MetaCore networks depending on statistical models, the correlation between certain MetaCore networks and one pathway was represented by a standardized similarity score (*SSscore*) for each mice line, which was defined as:

$${\text{SSscore}}_{i}\text{ of SAM}=\frac{1}{n_{1}}\sum_{j=1}^{n_{1}}\text{similarity score of}\;({\text{SAMNet}}_{j},{\text{Pathway}}_{i})$$

$${\text{SSscore}}_{i}\text{ of PrP}=\frac{1}{n_{1}}\sum_{j=1}^{n_{1}}\text{similarity score of}\;({\text{PrPNet}}_{j},{\text{Pathway}}_{i})$$

Where *i* = 1, 2, …, 93, corresponding to 93 KEGG pathways *n*_1_=n_2_=3, corresponding to three different size MetaCore networks of each mice strain, SAM corresponding to SAMP8 mice, and PrP corresponding to APP/PS1 mice. The *SSscores* were calculated for 93 pathways. *SSscore* of each mice by each of 93 KEGG pathways were listed in Supplemental material 4.

### Statistical analysis

All data are expressed as mean ± SEM. GraphPad Prism^®^, version 6 was used to plot and analyze data. Data between two groups were compared by Student's *t*-test. Comparisons of data from multiple groups against one group was analyzed by one-way analysis of variance (ANOVA) followed by Dunnett's post hoc test or two-way repeated-measures analysis of variance with Tukey multiple comparisons test. *P* < 0.05 was taken as statistically significant. Linear regression was using two-tailed Pearson analysis with 95% confidence interval by GraphPad Prism, the correlation was considered statistically significant if *P* < 0.05.

## SUPPLEMENTARY MATERIALS












