# Supplementary material for: Neuroendocrine immunomodulation network dysfunction in SAMP8 mice and PrP-hAβPPswe/PS1ΔE9 mice: potential mechanism underlying cognitive impairment
Source: Oncotarget. 2016 Mar 28;7(17):22988–3005. doi: 10.18632/oncotarget.8453 (PMC5029605; doi:10.18632/oncotarget.8453)
Supplement: Supplementary file 5 [file oncotarget-07-22988-s005.pdf]

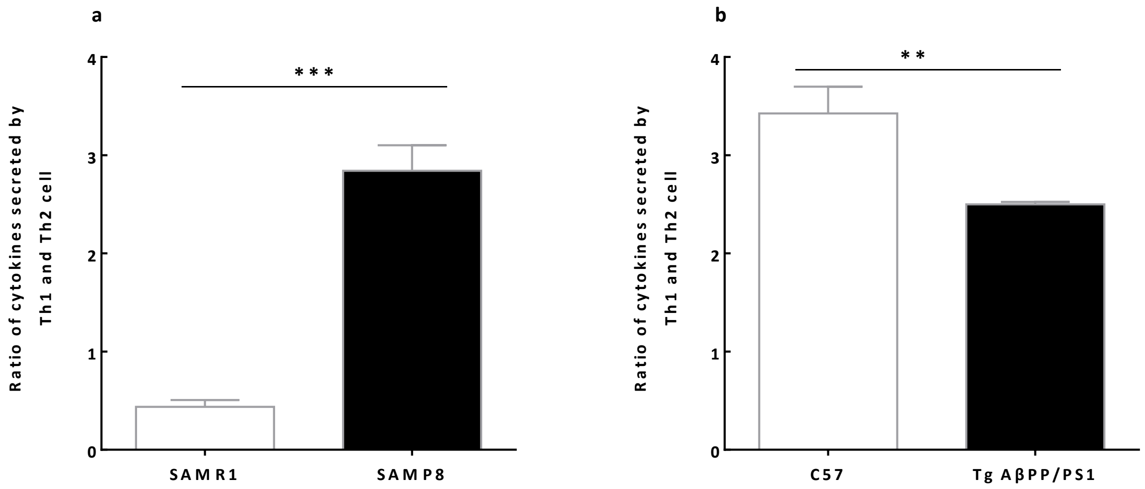

Supplemental material 5 The imbalance between Th1 and Th2 cell contributing to cognitive impairment in SAMP8 mice and PrP-hAβPPswe/PS1$^{\Delta E9}$ mice. (a) The imbalance between Th1 and Th2 cell in SAMP8 mice. (b) The imbalance between Th1 and Th2 cell in PrP-hAβPPswe/PS1$^{\Delta E9}$ mice. Multiple linear regression analysis showed that IL-2, TNF-β secreted by Th1 cell, IL-5 secreted by Th2 cell, GM-CSF secreted by both Th1 and Th2 cell contributed to the cognitive impairment of SAMP8 mice. And IFN-γ, TNF-β secreted by Th1 cell, GM-CSF secreted by both Th1 and Th2 cell contributed to the cognitive impairment of PrP-hAβPPswe/PS1$^{\Delta E9}$ mice. The data normalization method for concentration of these cytokines was $\frac{x-Min}{Max+Min}$, *x* was the concentration of cytokine, *Min* was minimum concentration in the group, *Max* was maximum concentration in the group. The ratio of cytokines secreted by Th1 cell and Th2 cell of SAMR1 and SAMP8 mice was calculated by $\frac{C_{IL-2}+C_{GM-CSF}+C_{TNF-\beta}}{C_{IL-5}+C_{GM-CSF}}$, and that of C57 and PrP-hAβPPswe/PS1$^{\Delta E9}$ mice was calculated by $\frac{C_{IFN-\gamma}+C_{GM-CSF}+C_{TNF-\beta}}{C_{GM-CSF}}$, *C* was abbreviation for concentration. $^{**}P<0.01$, $^{***}P<0.001$, compared with control mice. Data represent mean ± SEM, n=10~15, unpaired Student T-test.
