# Supplementary material for: Neuroendocrine immunomodulation network dysfunction in SAMP8 mice and PrP-hAβPPswe/PS1ΔE9 mice: potential mechanism underlying cognitive impairment
Source: Oncotarget. 2016 Mar 28;7(17):22988–3005. doi: 10.18632/oncotarget.8453 (PMC5029605; doi:10.18632/oncotarget.8453)
Supplement: Supplementary file 6 [file oncotarget-07-22988-s006.pdf]

Supplemental material 6 The deficiency of NIM network in PrP-hAβPPswe/PS1$^{\Delta E9}$ mice

Indictor	Model	Age(month)	Gender	Site	Feature	Reference
Hypothalamic-pituitary-adrenal (HPA) axis						
CRH	Tg2576	12	Male	Hypothalamus	Increase	[1]
		18	Male	Hypothalamus	Not significant	[1]
		24	Male	Hypothalamus	Decrease	[1]
CORT	PrP-hAβPPswe/PS1$^{\Delta E9}$	8	Male	Plasma	Increase	[2]
	Tg2576	6.75	Male and Female	Plasma	Increase	[3]
		4	Female	Serum	Increase	[4]
	PS1$_{M146V}$/APPswe/ tau$_{P301L}$	7	Male	Plasma	Not significant	[5]
		14	Male	Plasma	Not significant	[5]
		20	Male	Plasma	Not significant	[5]
		15	Male	Plasma	Increase	[6]
		9	Male	Plasma	Increase	[7]
		12	Male	Plasma	Increase	[7]
		15	Male	Plasma	Increase	[7]
		18	Male	Plasma	Increase	[7]
Hypothalamus-pituitary-gonadal (HPG) axis						
T	TASTPM	12.5	Male	Plasma	Decrease	[8]
Proinflammatory factor						
Interleukin						
IL-1β	PrP-hAβPPswe/PS1$^{\Delta E9}$	9	Female	Serum	Increase	[9]
		7	-	Hippocampal and Cortex	Increase	[10]
		9	Male	Brain	Increase	[11]
		9	Male	Brain	Increase	[12]
		12	Female	Cortex	Increase	[13]
		9.5	Male	Cortex	Increase	[14]
		8~9	Female and Male	Hippocampal and Cortex	Increase	[15]
		7	-	Cortex	Increase	[16]
	APP V717I	10	-	Cortex	Increase	[17]
	APP$_{751SL}$ /PS1$_{M146L}$	6	Male	Microglial cell	Increase	[18]
		18	Male	Microglial cell	Increase	[18]

Indictor	Model	Age(month)	Gender	Site	Feature	Reference
	$APP_{K670N/M671L}/PS1_{M146V}$	14.5	-	Plasma	Increase	[19]
IL-2	$APP_{K670N/M671L}/PS1_{M146V}$	14.5	-	Plasma	Not significant	[19]
	$APP_{K670N/M671L}/PS1_{M146L}$	11	-	Plasma	Increase	[20]
IL-6	PrP-hAβPPswe/$PS1^{\Delta E9}$	9	Male	Brain	Increase	[12]
		15-24	Male or female	Serum	Increase	[21]
		9	Female	Serum	Increase	[22]
		6	Female	Serum	Not significant	[23]
		9	Female	Serum	Increase	[23]
		12	Female	Serum	Increase	[23]
		9	-	Hippocampus	Increase	[24]
		14	-	Hippocampus	Increase	[24]
	$APP_{K670N/M671L}/PS1_{M146V}$	14.5	-	Plasma	Increase	[19]
	APPswe/PS1-A246E	12	Male	Brain	Increase	[25]
IL-17	$APP_{K670N/M671L}/PS1_{M146V}$	14.5	-	Plasma	Not significant	[19]
Colony stimulating factor						
GM-CSF	PrP-hAβPPswe/$PS1^{\Delta E9}$	9	Female	Serum	Increase	[9]
	$APP_{K670N/M671L}/PS1_{M146V}$	14.5	-	Plasma	Not significant	[19]
		11	-	Plasma	Increase	[20]
Interferon						
IFN-γ	PrP-hAβPPswe/$PS1^{\Delta E9}$	15-24	Male or female	Serum	Increase	[21]
	$APP_{K670N/M671L}/PS1_{M146V}$	14.5	-	Plasma	Not significant	[19]
	$APP_{K670N/M671L}/PS1_{M146L}$	11	-	Plasma	Increase	[20]
Tumor necrosis factor						
TNF-α	PrP-hAβPPswe/$PS1^{\Delta E9}$	7	-	Hippocampal and Cortex	Increase	[10]
		9	Male	Brain	Increase	[11]
		10	Male	Brain	Increase	[12]
		9.5	Male	Cortex	Increase	[14]
		9	Female	Serum	Increase	[22]
		6	Female	Serum	Not significant	[23]
		9	Female	Serum	Increase	[23]
		12	Female	Serum	Increase	[23]
		9	-	Hippocampus	Not significant	[24]

Indictor	Model	Age(month)	Gender	Site	Feature	Reference
		14	-	Hippocampus	Increase	[24]
		7	-	Cortex	Increase	[16]
	$PS1_{M146V}$/APPswe/ tau_{P301L}	6	Male	Hippocampus	Increase	[26]
	$APP_{K670N/M671L}$/$PS1_{M146V}$	14.5	-	Plasma	Not significant	[19]
	$APP_{K670N/M671L}$/$PS1_{M146L}$	11	-	Plasma	Increase	[20]
	APPswe/PS1-A246E	12	Male	Brain	Increase	[25]
	APP_{751SL} /$PS1_{M146L}$	18	Male	Microglial cell	Increase	[18]
	APP V717I	10	-	Cortex	Increase	[17]
Chemotactic factor						
MCP-1	PrP-hAβPPswe/$PS1^{\Delta E9}$	4.5	Male or female	Brain	Not significant	[27]
		6.5	Male or female	Brain	Not significant	[27]
		12.5	Male or female	Brain	Increase	[27]
		9	-	Hippocampus	Not significant	[24]
		14	-	Hippocampus	Not significant	[24]
MIP-1β	$APP_{K670N/M671L}$/$PS1_{M146V}$	14.5	-	Plasma	Increase	[19]
Eotaxin	$APP_{K670N/M671L}$/$PS1_{M146V}$	14.5	-	Plasma	Not significant	[19]
RANTES	$APP_{K670N/M671L}$/$PS1_{M146V}$	14.5	-	Plasma	Not significant	[19]
Antiinflammatory factor						
IL-4	PrP-hAβPPswe/$PS1^{\Delta E9}$	9	Female	Serum	Not significant	[9]
	$APP_{K670N/M671L}$/$PS1_{M146V}$	14.5	-	Plasma	Increase	[19]
	$APP_{K670N/M671L}$/$PS1_{M146L}$	11	-	Plasma	Increase	[20]
IL-5	$APP_{K670N/M671L}$/$PS1_{M146V}$	14.5	-	Plasma	Increase	[19]
IL-10	PrP-hAβPPswe/$PS1^{\Delta E9}$	9	Female	Serum	Not significant	[9]
		9.5	Male	Cortex	Not significant	[14]
	$APP_{K670N/M671L}$/$PS1_{M146V}$	14.5	-	Plasma	Increase	[19]
G-CSF	$APP_{K670N/M671L}$/$PS1_{M146V}$	14.5	-	Plasma	Not significant	[19]

1. Horgan J, Miguel-Hidalgo JJ, Thrasher M and Bissette G. Longitudinal brain corticotropin releasing factor and somatostatin in a transgenic mouse (TG2576) model of Alzheimer's disease. Journal of Alzheimer's disease : JAD. 2007; 12(2):115-127.

2. Sierksma AS, van den Hove DLA, Rutten K, Chouliaras L, Rostamian S, Steinbusch HWM and Prickaerts J. Chronic phosphodiesterase type 2 inhibition improves spatial memory and alters synaptic density in the hippocampus in the APPswe/PS1dE9 mouse model of Alzheimer's disease. Society for Neuroscience Abstract Viewer and Itinerary Planner. 2011; 41.

3. Dong H, Yuede CM, Yoo HS, Martin MV, Deal C, Mace AG and Csernansky JG. Corticosterone and related receptor expression are associated with increased beta-amyloid plaques in isolated Tg2576 mice. Neuroscience. 2008; 155(1):154-163.

4. Carroll JC, Iba M, Bangasser DA, Valentino RJ, James MJ, Brunden KR, Lee VM and Trojanowski JQ. Chronic stress exacerbates tau pathology, neurodegeneration, and cognitive performance through a corticotropin-releasing factor receptor-dependent mechanism in a transgenic mouse model of tauopathy. The Journal of neuroscience : the official journal of the Society for Neuroscience. 2011; 31(40):14436-14449.

5. Hohsfield LA, Daschil N, Oradd G, Stromberg I and Humpel C. Vascular pathology of 20-month-old hypercholesterolemia mice in comparison to triple-transgenic and APPSwDI Alzheimer's disease mouse models. Molecular and cellular neurosciences. 2014; 63:83-95.

6. Gimenez-Llort L, Arranz L, Mate I and De la Fuente M. Gender-Specific Neuroimmunoendocrine Aging in a Triple-Transgenic 3xTg-AD Mouse Model for Alzheimer's Disease and Its Relation with Longevity. Neuroimmunomodulation. 2008; 15(4-6):331-343.

7. Green KN, Billings LM, Roozendaal B, McGaugh JL and LaFerla FM. Glucocorticoids increase amyloid-beta and tau pathology in a mouse model of Alzheimer's disease. The Journal of neuroscience : the official journal of the Society for Neuroscience. 2006; 26(35):9047-9056.

8. Hu ZP, Browne ER, Liu T, Angel TE, Ho PC and Chan EC. Metabonomic profiling of TASTPM transgenic Alzheimer's disease mouse model. Journal of proteome research. 2012; 11(12):5903-5913.

9. Danielyan L, Klein R, Hanson LR, Buadze M, Schwab M, Gleiter CH and Frey WH, II. Protective Effects of Intranasal Losartan in the APP/PS1 Transgenic Mouse Model of Alzheimer Disease. Rejuvenation Research. 2010; 13(2-3):195-201.

10. Xuan A-G, Pan X-B, Wei P, Ji W-D, Zhang W-J, Liu J-H, Hong L-P, Chen W-L and Long D-H. Valproic Acid Alleviates Memory Deficits and Attenuates Amyloid-beta Deposition in Transgenic Mouse Model of Alzheimer's Disease. Molecular Neurobiology. 2015; 51(1):300-312.

11. Lv C, Wang L, Liu X, Yan S, Yan SS, Wang Y and Zhang W. Multi-faced neuroprotective effects of geniposide depending on the RAGE-mediated signaling in an Alzheimer mouse model. Neuropharmacology. 2015; 89:175-184.

12. Song Y, Cui T, Xie N, Zhang X, Qian Z and Liu J. Protocatechuic acid improves cognitive deficits and attenuates amyloid deposits, inflammatory response in aged A beta PP/PS1 double transgenic mice. International Immunopharmacology. 2014; 20(1):276-281.

13. Yan J-J, Jung J-S, Kim T-K, Hasan MA, Hong C-W, Nam J-S and Song D-K. Protective Effects of Ferulic Acid in Amyloid Precursor Protein Plus Presenilin-1 Transgenic Mouse Model of Alzheimer Disease. Biological & Pharmaceutical Bulletin. 2013; 36(1):140-143.

14. Cherry JD, Liu B, Frost JL, Lemere CA, Williams JP, Olschowka JA and O'Banion MK. Galactic Cosmic Radiation Leads to Cognitive Impairment and Increased A beta Plaque Accumulation in a Mouse Model of Alzheimer's Disease. Plos One. 2012; 7(12).

15. Gallagher JJ, Minogue AM and Lynch MA. Impaired Performance of Female APP/PS1 Mice in the Morris Water Maze Is Coupled with Increased A beta Accumulation and Microglial Activation. Neurodegenerative Diseases. 2013; 11(1):33-41.

16. Cheng Y, Dong Z and Liu S. beta-Caryophyllene Ameliorates the Alzheimer-Like Phenotype in APP/PS1 Mice through CB2 Receptor Activation and the PPAR gamma Pathway. Pharmacology. 2014; 94(1-2):1-12.

17. Jiang H, Liu CX, Feng JB, Wang P, Zhao CP, Xie ZH, Wang Y, Xu SL, Zheng CY and Bi JZ. Granulocyte colony-stimulating factor attenuates chronic neuroinflammation in the brain of amyloid precursor protein transgenic mice: an Alzheimer's disease mouse model. The Journal of international medical research. 2010; 38(4):1305-1312.

18. Jimenez S, Baglietto-Vargas D, Caballero C, Moreno-Gonzalez I, Torres M, Sanchez-Varo R, Ruano D, Vizuete M, Gutierrez A and Vitorica J. Inflammatory Response in the Hippocampus of PS1(M146L)/APP(751SL) Mouse Model of Alzheimer's Disease: Age-Dependent Switch in the Microglial Phenotype from Alternative to Classic. Journal Of Neuroscience. 2008;

28(45):11650-11661.
19. Sanchez-Ramos J, Song S, Sava V, Catlow B, Lin X, Mori T, Cao C and Arendash GW. Granulocyte Colony Stimulating Factor Decreases Brain Amyloid Burden And Reverses Cognitive Impairment In Alzheimer's Mice. Neuroscience. 2009; 163(1):55-72.
20. Cao C, Arendash GW, Dickson A, Mamcarz MB, Lin X and Ethell DW. Abeta-specific Th2 cells provide cognitive and pathological benefits to Alzheimer's mice without infiltrating the CNS. Neurobiol Dis. 2009; 34(1):63-70.
21. Barrett JP, Minogue AM, Jones RS, Ribeiro C, Kelly RJ and Lynch MA. Bone Marrow-Derived Macrophages from A beta PP/PS1 Mice are Sensitized to the Effects of Inflammatory Stimuli. Journal Of Alzheimers Disease. 2015; 44(3):949-962.
22. Wang T, Jiang Y, Xiao L, 江毅 and 肖联平. Expression of amyloid beta-protein in bone tissue of APP/PS1 transgenic mouse. National Medical Journal of China. 2013; 93(1):65-68.
23. Yang M-W, Wang T-H, Yan P-P, Chu L-W, Yu J, Gao Z-D, Li Y-Z and Guo B-L. Curcumin improves bone microarchitecture and enhances mineral density in APP/PS1 transgenic mice. Phytomedicine. 2011; 18(2-3):205-213.
24. Schmole AC, Lundt R, Ternes S, Albayram O, Ulas T, Schultze JL, Bano D, Nicotera P, Alferink J and Zimmer A. Cannabinoid receptor 2 deficiency results in reduced neuroinflammation in an Alzheimer's disease mouse model. Neurobiol Aging. 2015; 36(2):710-719.
25. Serrano J, Fernandez AP, Martinez-Murillo R and Martinez A. High sensitivity to carcinogens in the brain of a mouse model of Alzheimer's disease. Oncogene. 2010; 29(15):2165-2171.
26. Giuliani D, Ottani A, Zaffe D, Galantucci M, Strinati F, Lodi R and Guarini S. Hydrogen sulfide slows down progression of experimental Alzheimer's disease by targeting multiple pathophysiological mechanisms. Neurobiology Of Learning And Memory. 2013; 104:82-91.
27. Jardanhazi-Kurutz D, Kummer MP, Terwel D, Vogel K, Thiele A and Heneka MT. Distinct Adrenergic System Changes And Neuroinflammation In Response To Induced Locus Ceruleus Degeneration In App/Ps1 Transgenic Mice. Neuroscience. 2011; 176:396-407.
